# Diversity of *Fusarium* Species Causing Storage Rot of Table Beet in the Moscow Region of the Russian Federation

**DOI:** 10.3390/jof12060413

**Published:** 2026-06-05

**Authors:** Svetlana Vetrova, Elena Kozar, Irina Engalycheva, Kseniya Mukhina, Vera Chizhik, Viktor Martynov

**Affiliations:** 1 Federal State Budgetary Scientific Institution Federal Scientific Vegetable Center (FSBSI FSVC), VNIISSOK, 143072 Moscow, Russia; kozar_eg@mail.ru (E.K.); engirina1980@mail.ru (I.E.); kseniyamukhina@yandex.ru (K.M.); chizhikvera@bk.ru (V.C.); 2 Federal State Budgetary Scientific Institution All-Russian Research Institute of Agricultural Biotechnology (FSBSI ARRIAB), 127550 Moscow, Russia; martynov.vik@gmail.com

**Keywords:** *Fusarium*, table beet, morphology, pathogenicity, phylogenetic analysis

## Abstract

*Fusarium* fungi are known to infect table beet (*Beta vulgaris* subsp. *vulgaris*) plants at various stages of development worldwide. *Fusarium* root rot, which develops post-harvest during long-term storage, is of particular economic significance. In Russia, there is no up-to-date information about the species diversity of pathogens causing this disease of table beets, which determined the purpose of this study. A total of 28 *Fusarium* isolates were collected from affected beet roots grown in the Moscow region of the Russian Federation from 2018 to 2023 years. Molecular phylogeny based on the *TEF-1α* and *RPB2* genes in combination with morphological characterization showed that five *Fusarium* species were involved in the pathogenesis of *Fusarium* root rot of table beet during storage: *F. acuminatum* (43% of the total number of isolates), *F. avenaceum*, *F. campestre* (FTSC); *F. sporotrichioides* (FSAMSC) and *F. solani* (FSSC). At the same time, the species *F. acuminatum, F. campestre*, and *F. sporotrichioides* were first discovered on beet root in the Russian Federation. Temperature sensitivity of the identified species was studied at 5 °C and 25 °C. According to the value of the cold sensitivity index (CTI) on the nutrient medium and native substrate, the isolates were distributed differently: *F. campestre* (0.32) > *F. acuminatum* (0.22) > *F. avenaceum* (0.21) > *F. sporotrichioides* (0.19) > *F. solani* (0.20) and *F. acuminatum* (0.32) > *F. campestre* (0.21) > *F. solani* (0.03) > *F. avenaceum* and F*. sporotrichioides* (0.01), respectively. This confirms the need to study the pathogenic properties of isolates on a natural substrate (host plant) under different temperature conditions. When infected with the dominant and most aggressive species *F. acuminatum*, there was a high variation in the size of the affected area, depending on the genotype of the lines, under both temperature conditions (Va = 2–8 mm^3^ at 5 °C and Va = 31–1760 mm^3^ at 25 °C). Therefore, this species can be considered to be the most objective differentiating factor in assessing the resistance of table beet roots to fusarium rot, which determines the need to include it in the breeding process for creating resistant varieties and hybrids for the Central region of Russia. The data obtained in this study are of great importance for developing strategies for managing *Fusarium* fungi associated with *Fusarium* rot of beet-root during storage. The research results will also be relevant for other vegetable crops that remain fresh for long periods of time or undergo vernalization in the case of seed production at low temperatures.

## 1. Introduction

Table beet (*Beta vulgaris* subsp. *vulgaris*) is a strategically important vegetable crop in the Russian Federation. This root crop is gaining increasing popularity each year due to its rich, unique nutrient composition [1]. Recently, table beet has been actively used in food production: in ready-made salads, frozen soups, chips, snacks, borscht dressings, smoothie powders, food colorings, microgreens, biological additives, and much more [2,3,4,5].

The special value and popularity of this crop is determined by its easy to grow, high yield and long shelf life in fresh form [6].

In the Russian Federation, table beet cultivation accounts for approximately 10% of all open-field crop areas—approximately 30,000 hectares. The main industrial production of table beet is concentrated in the Central, Southern, and Volga Federal Districts, where approximately 40% of the gross harvest is grown. The leaders in terms of table beet production in recent years are Moscow (68.74 thousand tons—18.0% of the gross harvest), Samara (32.47 thousand tons—8.5%), Rostov (18.81 thousand tons—4.9%), Volgograd (16.24 thousand tons—4.3%) and Omsk (16.45 thousand tons—4.3%) regions [7]. The average yield of table beets in the country is about 30 tons/ha. In farms in the Moscow, Leningrad and Volgograd regions with a high level of development of industrial technologies, the yield reaches 70–80 tons/ha [8].

Table beet is susceptible to various fungal and bacterial pathogens both during plant growth and during root storage. The most harmful of these are *Fusarium* spp., *Phoma* spp., *Alternaria radicina*, *Sclerotinia sclerotiorum*, *Rhizoctonia solani Kuhn*, *Pythium ultimum Trow*, *Cercospora beticola Sacc*, and *Pseudomonas syringae* pv. *aptata* [9,10,11,12,13,14,15,16].

In the Moscow region of the Russian Federation, annual monitoring of table beet roots after long-term storage (October–April) revealed that significant damage to the yield is caused by *Fusarium* root rot pathogens [17]. Localization of Fusarium fungi in the root head area leads to rotting and death of the central bud, and often the entire root head, making it impossible for flower stalks to grow and produce seed.

Information about the species diversity of *Fusarium* fungi that cause *Fusarium* root rot of table beets is currently very limited. We were able to find only a few publications devoted to this issue. It is reported that in the 1980s in New York State, several *Fusarium* species were isolated and identified from infected root tissue and stems of plants grown in fields and greenhouses. There were *F. roseum*, *F. solani*, *F. oxysporum* and *F. moniliforme*. Pathogenicity testing showed that all isolates were non-pathogenic to beet plants and affected only roots already infected with other diseases [18]. Later in 2017, a group of scientists, also in New York State, conducted phytosanitary monitoring of beet crops and found that, among other root crop pathogens, internal dry rot caused by *Fusarium* spp. was the predominant one [9].

Specifically, in New York State in the 1970s, *F. oxysporum*, *F. roseum*, *F. solani* and *F. monoliforme* were isolated and identified from infected roots and stems [14]. In the Russian Federation, also at the end of the 20th century, scientists from the FSBSI FSVC, based on a study of the morphological characteristics of colonies of isolated pathogens, identified the following spectrum of *Fusarium* species in this crop. In the Moscow region, the pathocomplex of *Fusarium* root rot of table beet included the species *F. sambucinum*, *F. culmorum*, *F. solani*, *F. sporotrichiella*, *F. oxysporum* and *F. avenaceum* [19,20].

Species identification of *Fusarium* fungi by analyzing their micromorphological and cultural characteristics is time-consuming and often difficult due to their similarity and high variability of a number of traits [21,22]. For reliable identification and confirmation of the taxonomic status of strains, molecular genetic methods are used. These methods are based on the analysis of nucleotide sequences of phylogenetically informative regions of the genome: the *EF1a* gene encoding translation elongation factor 1 alpha, the *RPB2* gene encoding the second-largest subunit of RNA polymerase II, and the *CYP51C* gene encoding sterol 14-demethylase. An integrated approach, using molecular markers and studying the morphological and biological characteristics of fungi, will enable to address various research challenges in understanding the biological diversity of the genus *Fusarium* and its geographic distribution at a new level [23,24]. This information is crucial for phytopathological research, a better understanding of the distribution and harmfulness of individual species, and the development of effective strategies to combat associated diseases [25,26].

Currently, data on the actual composition and aggressiveness of the *Fusarium* spp. pathocomplex causing *Fusarium* rot of table beet roots during storage are lacking in Russia. Therefore, the aim of this study was to identify the species of *Fusarium* fungi associated with storage root rot in the Moscow region of Russia and to assess their pathogenicity on the host plant.

## 2. Materials and Methods

### 2.1. Weather Conditions During the Growing Seasons in the Years of Research

**Figure 1:**
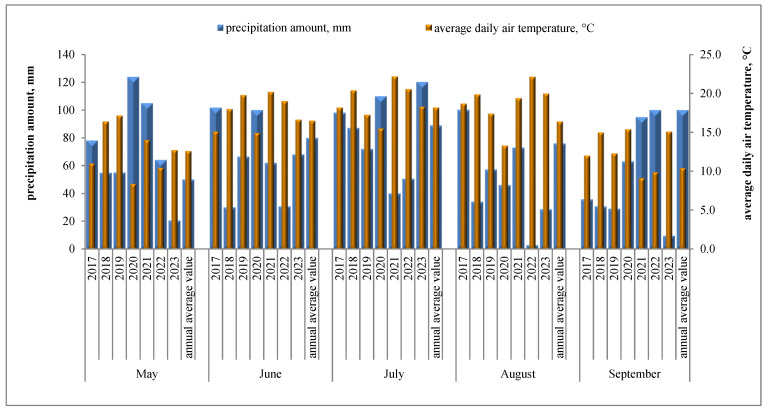
Average daily air temperature and precipitation amount in the Moscow region by month during the growing season in the years of research (according to the hydrometeorological station “Nemchinovka” in the Odintsovo district of the Moscow region. Station coordinates: 55.707902, 37.368398).

Average daily air temperature and precipitation amount in the Moscow region by month during the growing season are presented since 2017, as the study results discuss the potential relationship between the prevalence of *Fusarium* rot during the storage period of table beet edible roots, depending on weather conditions during the growing season (Figure 1).

**Figure 2:**
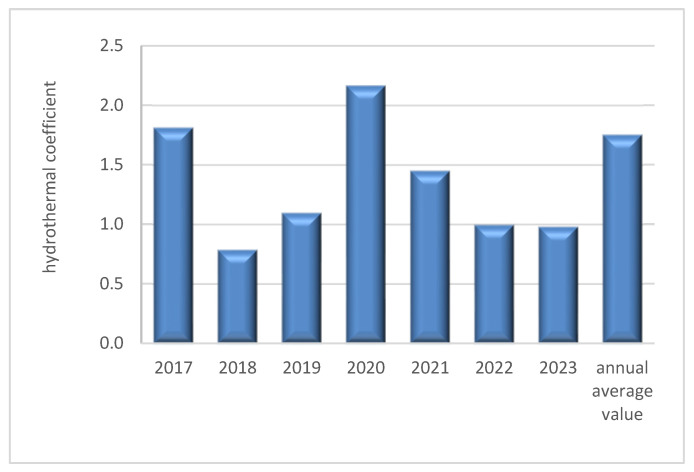
Hydrothermal coefficient during the growing seasons in the years of research (Moscow region).

The growing season weather conditions in 2017 and 2020 were identical and characterized by cool and humid summers (HTC = 1.9 and 2.2, respectively) (Figure 2). Average air temperatures during the growing season were 15.1 °C and 13.3 °C, respectively, which is 1.4–3.2 °C below the long-term average. The growing seasons of 2018, 2019, 2021 and 2022 were characterized as warm and dry (HTC = 0.8–1.4). Average air temperatures during the growing season were 1.6–2.5 °C higher than the long-term average, and 3–4 °C higher in the summer months, with precipitation deficit (60–70% of normal). The 2023 growing season was also warm and dry (HTC = 1.0), but was characterized by uneven precipitation, with minimal amounts in May and August (40% of the normal). In July, precipitation exceeded the long-term average by 36%. Air temperatures during the growing season averaged 1.7 °C above the long-term average. Significantly higher temperatures than the long-term average were observed at the end of the growing season in August-September, by 3.6 °C and 4.7 °C, respectively, with virtually no precipitation.

### 2.2. Phytopathological Monitoring of the Prevalence of Fusarium Storage Rot on Table Beet Crops in the Moscow Region

The research was conducted from 2017 to 2023. The roots of selected beet varieties were grown in the experimental fields of the main crop rotation of the Federal State Budgetary Scientific Institution Federal Scientific Vegetable Center (FSBSI FSVC) in the Moscow Region of the Russian Federation, using the standard technology [27]. In total, 350 samples of root crops were grown over the years of research. Each sample contained between 30 and 120 root crops.

During the harvest, each sample was selected for marketable root crops without signs of disease, placed in separate vegetable nets, and stored in containers with polyethylene liners in a vegetable storage facility. During the storage period from October to December, the storage temperature was 2–3 °C, and then from January to April, it was 4–6 °C. The air humidity was 90–92% throughout the storage period.

From 2018 to 2023, after storage of the beet samples selected for the breeding table, phytosanitary monitoring of edible roots for *Fusarium* rot was conducted. All stored root vegetables were examined. About 3500 root vegetables were analyzed every year. The pathogen’s localization was taken into account during visual diagnostics of disease symptoms.

For each sample, the percentage of disease prevalence and the disease severity index were calculated using the following formulas:

(1) $$Percent\;disease\;prevalence=\frac{Total\;number\;of\;diseased\;plants}{Total\;number\;of\;examined\;plants}\times 100$$

(2) $$Disease\;severity\;index\left(DSI\right)=\frac{\sum \left(ScoreAmountofplants\times Correspondingscore\right)}{Maximumscore*Totalnumberofplants}*100$$

The Disease severity index was determined according to a seven-point scale: 0—no symptoms; 0.5—up to 5% of the surface is affected; 1—6–20% is affected; 2—21–50% is affected; 3—51–70% is affected; 4—more than 70% of the entire surface is affected; 5—the edible root is completely rotten [28].

### 2.3. Collection of Plant Material and Isolation of Fungi

Every year, during spring analysis and phytopathological examination of breeding samples, root crops with symptoms of Fusarium rot were selected.

Edible roots having various symptoms of damage were photographed in the laboratory, thoroughly rinsed under tap water, then surface sterilized by immersion in a 50% solution of commercial bleach «Belizna», containing 10–12% sodium hypochlorite for 5 min, followed by two rinses in sterile water. To isolate fungi at the junction of diseased and healthy areas, tissue was cut into several small segments, surface sterilized in 70% ethanol, rinsed in sterile water, dried, placed on potato dextrose agar (PDA) and Czapek-Dox medium, and kept at 25 °C for 3–5 days. Each colony grown was transferred by the hyphal tip to a new medium to obtain a pure isolate culture, followed by single-spore colonies. A total of 32 isolates of the genus *Fusarium* were isolated over the years of research.

### 2.4. DNA Extraction, PCR Amplification and Phylogenetic Analysis

The air mycelium of *Fusarium* fungi was collected from Petri dishes and placed into 1.5 mL Eppendorf tubes. The DNeasy Plant Pro Kit (QIAGEN, Hilden, Germany) was used to isolate DNA. The data on DNA purity and concentration were obtained using a NanoDrop device (Thermo Fisher Scientific, Waltham, MA, USA).

Primer sequences used for PCR amplification of ITS, tef1 and rpb2 loci and PCR conditions were described previously [29] and are now given in Table 1.

The composition of the PCR mixture was as follows: 2.5 μL of 10X PCR buffer, 1–10 ng of genomic DNA, 2.5 μL of 2.5 M dNTP, 10 pmol of each primer, 1 unit of Taq DNA polymerase (Syntol, Moscow, Russia) or Pfu DNA polymerase (Evrogen, Moscow, Russia), and sterile water to a volume of 25 μL. Amplification was performed in an MJ PTC-200 thermal cycler (Bio-Rad, Hercules, CA, USA). The amplification program consisted of one cycle of 94 °C for 3 min; 35 cycles of 94 °C for 30 s, for 30 s and 72 °C for 1 min; and a final extension at 72 °C for 5 min. PCR products were separated in agarose gel using the horizontal electrophoresis chamber Sub-Gell GT System and the PowerPac HC power supply (BioRad, Hercules, CA, USA). Visualization of the results was carried out using a GelDoc XR+ System transilluminator (Bio Rad, Hercules, CA, USA). Excision of PCR products from the gel was performed on an ECX-M transilluminator (VilberLourmat, Eberhardzell, Germany). The samples were purified with a ColGen kit (Syntol, Russia) and sequenced at Syntol. Species identification was carried out in GenBank NCBI (https://www.ncbi.nlm.nih.gov/genbank/ (accessed on 4 April 2026)) and FUSARIOID-ID database—Food, Fibre & Health (https://www.fusarium.org/).

Phylogenetic analysis was carried out using the Maximum Likelihood method and the Tamura-Nei model. The percentage of trees in which the associated taxa clustered together is shown next to the branches. Evolutionary analyses were performed in MEGA 12 [24].

### 2.5. Macro- and Micromorphology of Fusarium Isolates

Micro- and macromorphological characteristics of single-spore cultures were assessed by growing fungi on potato dextrose agar (PDA) medium. Isolates were cultured for 26 days under alternating light conditions (16 h day and 8 h night) at 25 °C. Colony morphology was characterized by the following features: aerial mycelium density and substrate mycelium (stroma) pigmentation. Microscopic characteristics of single-spore cultures were examined and recorded using a Zeiss Axio Lab A1 microscope (Carl Zeiss, Oberkochen, Germany) and ADF Image Capture software (version x64, 4.11.21522.20221011). At least 30–40 microstructures (conidia, chlamydospores) were measured for each isolate. The taxonomic status of *Fusarium* fungi was characterized using identification guides [30,31] and scientific publications.

### 2.6. Pathogenicity Test

To assess the pathogenicity of *Fusarium* isolates associated with storage rot of root crops and to confirm Koch’s postulates, four widely cultivated table beet varieties bred by FSBSI FSVC—Marusya, Krasny barkhat, Dobrynya, and Lyubava—were used. These varieties differ in their resistance to storage rot. First, all 32 isolates collected from affected edible roots were analyzed, followed by defined isolates of the five identified *Fusarium* species.

The edible roots were surface sterilized by immersion in a 50% sodium hypochlorite solution for 5 min, then rinsed twice in sterile water. After sterilization, the roots were cut into identically sized disks (4 × 3 × 1 cm) and placed in plastic containers (five replicates for five roots of each variety). Inoculation was carried out with mycelial blocks of a ten-day-old *Fusarium* fungal culture, placing them in the center of the disks. A sterile agar block served as the control. Containers with inoculated disks were moistened with sterile water and placed in the dark for two days, then in a light installation with alternating illumination (16 h day and 8 h night) at a temperature of 25 °C. The degree of damage was assessed on the seventh day after inoculation, measuring the diameter and depth of the affected area. The volume of the affected area (Va, cm^3^) was calculated using the formula for the volume of a cylinder, measuring the radius and depth of the affected area:

(3) $$Volume\;of\;the\;affected\;area\left(Va\right)=\pi *{radius\;of\;the\;affected\;area}^{2}*depth\;of\;the\;affected\;area$$

Based on the volume of the affected area of root disks, fungal isolates were differentiated into the following pathogenicity categories:

Non-pathogenic (NP): Va = 0 mm^3^.

Weakly aggressive (WA): Va = 1–70 mm^3^.

Moderately aggressive (MA): Va = 71–150 mm^3^.

Highly aggressive (HA): Va = ≥151 mm^3^.

### 2.7. Growth of Fusarium Species on Artificial Medium and Natural Substrate (Beet Root Disks) at Different Temperatures (Temperature Sensitivity) In Vitro

Eight isolates of five identified *Fusarium* species were grown on artificial medium (PDA) for 26 days under alternating light conditions (16 h day and 8 h night) at different temperatures (25 °C and 5 °C). Colony morphology, pigmentation, and fungal growth rate were assessed daily, recording the time of growth onset (the appearance of mycelium beyond the root disk). Colony diameter was measured in two transverse directions for three parallel inoculations (*n* = 3) until the plate was completely covered.

Inoculation of root disks of 13 table beet breeding lines (Nos. 174, 179, 184, 185, 187, 189, 204, 205, 206, 207, 208, 209, and 210) was carried out with 5 defined isolates of different *Fusarium* species. Five edible roots of each line were taken for inoculation in triplicate. Artificial inoculation of disks was carried out in the same way as in the pathogenicity test. The experiment was set up at two temperatures: 25 °C and 5 °C. The results were recorded on the tenth day after inoculation. The lines were differentiated into resistant (Va^25 °C^ = 1–50 mm^3^; Va^5 °C^ = 0 mm^3^); relatively resistant (Va^25 °C^ = 51–200 mm^3^, Va^5 °C^ = 0.1–5 mm^3^); moderately susceptible (Va^25 °C^ = 201–1000 mm^3^, Va^5 °C^ = 5–8 mm^3^) and susceptible (Va^25 °C^ > 1000 mm^3^).

Temperature sensitivity was determined according to the cold tolerance index (CTI) of linear growth using the formula:

(4) $$CTI\left(Temperature\;sensitivity\right)=\frac{D5\left(colony\;diameter\;et\;25\;{}^{\circ}C\;on\;the\;10th\;day\right)}{D25\left(colony\;diameter\;et\;5\;{}^{\circ}C\;on\;the\;10th\;day\right)},$$

The higher the CTI (closer to 1), the better the pathogen is relatively adapted to cold.

### 2.8. Statistical Analysis

Experimental data analysis and statistical evaluation were performed using Microsoft Excel 2010 and Statistica 10.0 software. To determine the significance of differences in the aggressiveness of *Fusarium* strains, one-way analysis of variance (ANOVA) was used with grouping of mean values into homogeneous groups according to Duncan’s multiple range test (*p* ≤ 0.05) [32].

## 3. Results

### 3.1. Phytopathological Monitoring and Symptoms

**Figure 3:**
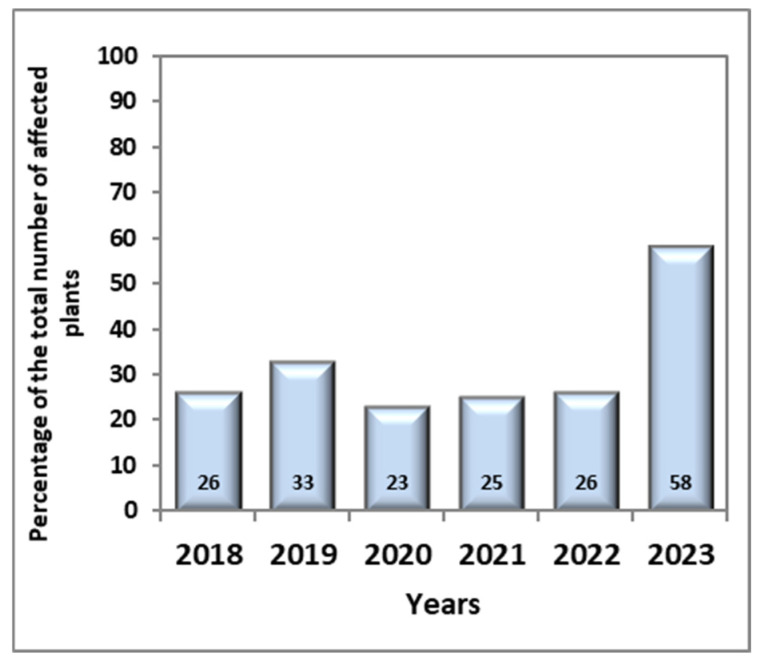
Prevalence of *Fusarium* rot on table beet roots after storage during the 2018–2023 study years (Moscow Region, Russian Federation).

Monitoring of disease development during storage of table beet roots grown in the Moscow region revealed that *Fusarium* fungi predominate in the pathocomplex causing the pit-storage decay. The percent of edible roots affected by *Fusarium* during storage varied across the study years, depending on the agroclimatic conditions of the year and the sample size analyzed, from 23% (2020) to 58% (2023) (Figure 3). The prevalence of Fusarium rot within individual varieties, depending on their resistance and the year of study, ranged from 3% to 70%. The disease severity index (DSI) averaged from 1.8 points (2020–2021) to 2.4 points (2019, 2023) (Table 2).

Warm and humid weather is believed to promote the active development of most *Fusarium* species. In our studies, a correlation analysis of the dependence of *Fusarium* rot prevalence on weather conditions during plant vegetation revealed the most consistent negative relationship between *Fusarium* rot development and the integral indicator—the hydrothermal coefficient (r = −0.42)—during the vegetation period before storage. As can be seen in Figure 1, the maximum number of edible roots affected by *Fusarium* rot during storage, relative to the number analyzed, was recorded in the 2018/2019 and 2022/2023 seasons, when the HTC during the vegetation periods of 2018 and 2022 was significantly lower than the long-term average—0.8 and 1.0, respectively. It can be assumed that the lack of soil moisture with persistently high daytime air temperatures during the period of intensive accumulation of dry matter and ripening of edible roots to the stage of technical maturity in these years provoked a decrease in the immune status of plants, which led to an increase in the intensity of spread in the field and the development of infection during storage.

**Figure 4:**
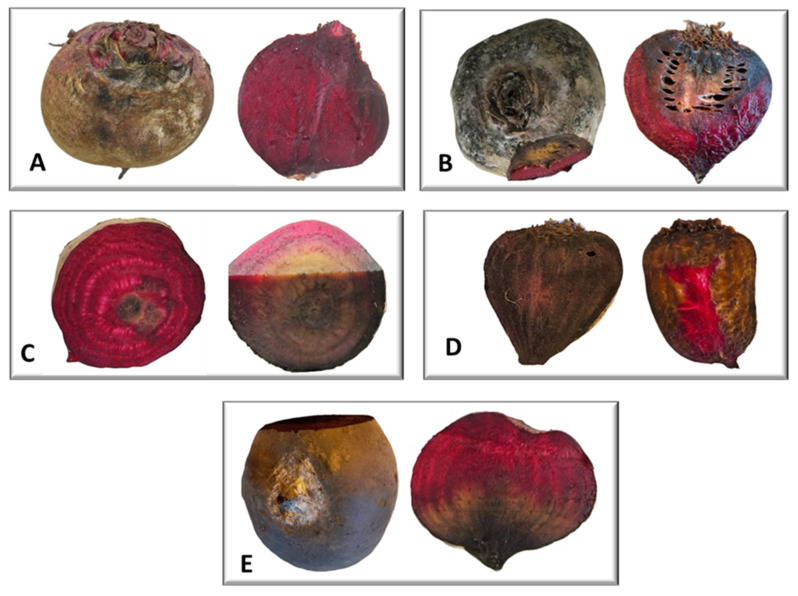
Symptoms of *Fusarium* rot development on table beet roots during in vivo storage (Moscow region, Russian Federation): (**A**)—ulcers on the surface; (**B**)—head; (**C**)—central part; (**D**)—entire root; (**E**)—tip.

When visually diagnosing *Fusarium* rot symptoms on edible roots, the location of pathogen development was taken into account, as this is a pivotal factor in the ability of *Fusarium*-infected roots to regrow and produce seed for the propagation of valuable breeding material. On average, over the years of research, in 33% of cases, *Fusarium* rot symptoms manifested as ulcers on the root surface, darkening and softening of the tissue in the affected area, sometimes with a small amount of exudate and a white-gray sporulation (Table 2 and Figure 4A). Signs of root head rot were observed in 30% of affected edible roots. In most cases, the disease severity index was high: the root head was completely affected and collapsed when pressed. When cutting the root, dry rot of the internal tissue spreading from the head was observed, forming cavities and a sporulation coating (Figure 4B). In 15% of roots without external surface damage, symptoms of *Fusarium* rot were detected only upon cutting the root, manifesting as rot of the internal tissue or conductive bundles (Figure 4C). At a high stage of disease development, when the DSI reached 3.5–4 points, complete damage of the entire root was observed (also 15% of the analyzed ones) (Figure 4D). In some roots (on average, 7% over the years of study), signs of damage were observed in the area of the tip and axial root in the form of dry rot without further spreading (Figure 4E). When planted, most of these edible roots took root and formed a leaf rosette.

In some years of research, a change in the dominant types of localization of symptoms of *Fusarium* root rot was observed. In the period 2018–2019, edible roots affected by *Fusarium* in the head area predominated (35–50% of all cases). This is typical for primary infection through the root neck or the development of a systemic infection. In the 2020–2023 years of research, a drastic increase in the proportion of roots with superficial ulcers (from 15 to 16% to 30–60%) was recorded, and a decrease in the proportion of lesions in the head area of the root to 10–32%. The proportion of completely affected roots reached 11–17% over five seasons, and 25% in 2018, indicating a high infectious load of vegetable agrocenosis with aggressive pathogens in relation to table beet roots (Table 2).

### 3.2. Isolation of Fusarium Isolates Associated with Storage Rot of Table Beet and Assessment of Their Aggressiveness

Fusarium isolates were isolated from different parts of Fusarium-infected beet roots during storage. Over the years of research, a total of 28 *Fusarium* isolates were obtained (Table 3), which were included in a study on species identification (morphological, molecular, and phylogenetic) and their phytopathogenic properties for the host plant.

A primary pathogenicity test on edible roots of two commercial beet varieties (Marusya and Krasny barkhat) with different field resistance to *Fusarium* under natural infection conditions showed that, based on the average disk damage zone after artificial inoculation with mycelial blocks of *Fusarium* isolates, the group of moderately aggressive isolates predominated (54%). The group of isolates characterized by high aggressiveness was the smallest (18%).

### 3.3. Phylogenetic Analysis of Fusarium Fungi

**Figure 5:**
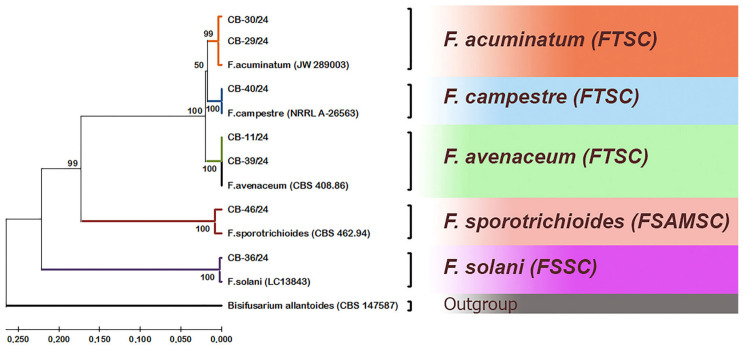
Phylogenetic analysis of *Fusarium* isolates based on the tef1a locus nucleotide sequences. The dendrogram was constructed in Mega 12 software using the UPGMA method, bootstrap—1000. The tef1 locus sequence from *Bisifusarium allantoides* was selected as the outgroup. Reference strain numbers from the FUSARIOID-ID database—Food, Fibre & Health are shown in parentheses.

Based on macro- and micromorphological characteristics, the isolates were preliminarily divided into five morphological groups with similar features. Representatives of each morphological group, each with varying levels of aggressiveness (a total of seven *Fusarium* isolates), were included in a study of species identification using molecular markers at three loci: ITS, tef1, and rpb2. The resulting nucleotide sequences of these loci in the studied isolates were compared with reference sequences from the curated FUSARIOID-ID database. As can be seen from the dendrogram, the sequences of all three analyzed loci were clustered with the reference sequences of the loci of the corresponding species, which made it possible to unambiguously identify the species of the strains studied (Figure 5).

As a result, strains F-CB-29 and F-sv-30 were discerned to belong to the species *F. acuminatum*—*F. tricinctum* species complex (FTSC). Strains F-CB-39, F-CB-11, according to a set of molecular and phenotypic characteristics, belong to the species *F. avenaceum* (FTSC); strain F-CB-40 belongs to the species *F. campestre* (FTSC); strain F-CB-46 belongs to the species *F. sporotrichioides*—*F. sambucinum* species complex (FSAMSC); strain F-CB-36 belongs to the species *F. solani*—*F. solani* species complex (FSSC). Thus, in the conditions of the Moscow region, five *Fusarium* species were identified as part of the pathocomplex of pathogens causing storage rot of table beet edible roots: *F. acuminatum*, *F. avenaceum*, *F. campestre*, *F. sporotrichioides*, and *F. solani*. *F. acuminatum* was the dominant species in the structure of the analyzed pathocomplex (43% of isolates from the total number), the percentage of other species was around 14%.

### 3.4. Morphology of Strains of Identified Fusarium Species

**Figure 6:**
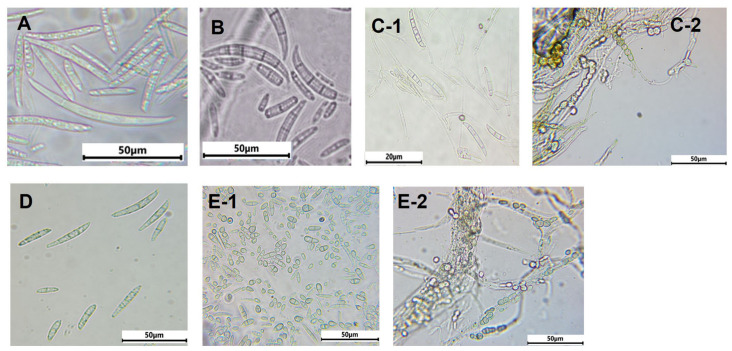
Micromorphological features of *Fusarium* species isolated from infected table beet roots (PDA at 25 °C, 16 h light/8 h dark, 26 days). (**A**)—*F. avenaceum* micro- and macroconidia; (**B**)—*F. acuminatum* micro- and macroconidia; (**C-1**)—*F. solani* micro- and macroconidia; (**C-2**)—*F. solani* chlamydospores; (**D**)—*F. campestre* micro- and macroconidia; (**E-1**)—*F. sporotrichioides* micro- and macroconidia; (**E-2**)—*F. sporotrichioides* chlamydospores.

Based on the morphological and cultural characteristics of colonies on artificial agar medium PDA, the analyzed isolates differed in the structure and density of the aerial mycelium, and pigment formation in the aerial mycelium and stroma (Table 4). The micromorphological characteristics of the *Fusarium* species involved in the pathocomplex causing storage rot of table beet roots are presented in Table 4 and Figure 6.

All species produced different proportions of macro- and microconidia of various shapes and sizes; chlamydospores were found only in the species *F. sporothrichioides* and *F. solani* (Table 4 and Figure 6).

### 3.5. Aggressiveness of Fusarium Species for Different Varieties of Table Beet Based on In Vitro Artificial Inoculation of Root Disks

**Figure 7:**
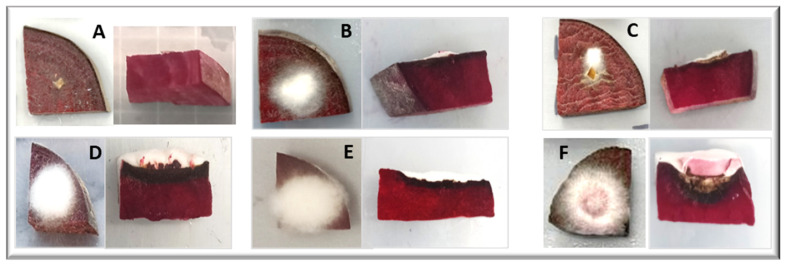
Symptoms of *Fusarium* rot on disks of table beet roots (for each letter, left photographic image—top view; right photographic image—cross-section) during artificial infection in vitro with identified *Fusarium* strains: (**A**) control (sterile agar block); (**B**) *F. avenaceum* (F-CB-39); (**C**) *F. sporotrichioides* (F-CB-46); (**D**) *F. campestre* (F-CB-40); (**E**) *F. solani* (F-CB-36); (**F**) *F. acuminatum* (F-CB-29).

Different *Fusarium* species caused different symptoms of beet root disk lesions (Figure 7). *F. avenaceum* and *F. sporotrichioides* strains were characterized by a small diameter zone and weak development of aerial mycelium on the disk surface, penetrating 1–2 mm into the tissue with a 1.5–2 times larger diameter of the affected area. The diameter of the affected area by *F. campestre* strain averaged 40–50% of the disk surface, forming a dense aerial mycelium in the center, beneath which a zone of darkening of the inner tissue layers reached 5–10 mm. By the seventh day, *F. solani* and *F. acuminatum* strains had almost completely occupied the entire disk surface, forming well-developed dense aerial mycelium, penetrating and mummifying internal tissue by 3–4 mm and 4–20 mm, respectively. At the same time, *F. acuminatum* in the center of the affected area caused destructive changes in the internal tissues with a change in their color, the formation of cavities and a coating of white mycelium.

Based on the average affected area volume, *F. acuminatum* demonstrated the greatest aggressiveness. When infected with this species, the average affected area volume on root disks across all analyzed varieties was 435 mm^3^, significantly exceeding the average affected area volume for isolates of other species. *F. solani* was the second most aggressive, with an average affected area volume of 303 mm^3^. This isolate demonstrated the most pronounced variety-specificity: from significant damage of root disks in the Dobrynya variety to mild damage in the Marusya and Lyubava varieties. The isolates of *F. sporotrichioides* and *F. campestre* were characterized by a moderate degree of aggressiveness according to the average value of the affected area, and the isolate of *F. avenaceum* showed weak aggressiveness towards the table beet roots of the analyzed varieties and did not differ significantly from the control (Table 5).

This experiment demonstrated varying degrees of resistance among the analyzed varieties to specific *Fusarium* species. For example, when the Krasny barkhat variety was infected, the degree of disk damage was minimal and did not differ significantly from the control. The Marusya variety was primarily affected by *F. acuminatum*, while the Dobrynya and Lyubava varieties showed varying degrees of susceptibility to the analyzed *Fusarium* species. Overall, the degree of damage and symptomatic manifestations in the table beet varieties after artificial inoculation corresponded to their field resistance.

Thus, the infection outcome after inoculation of root disks with different isolates was influenced by both the aggressiveness of the analyzed *Fusarium* species and the specific resistance of the cultivars. A two-way analysis of variance revealed the strongest interaction effect of both factors, accounting for 47%, as well as a significant effect of the *Fusarium* species, accounting for 45% of the total variance. The genotype effect was also significant, accounting for 8%.

### 3.6. In Vitro Growth of Fusarium Species on Artificial Nutrient Media at Different Temperatures

**Figure 8:**
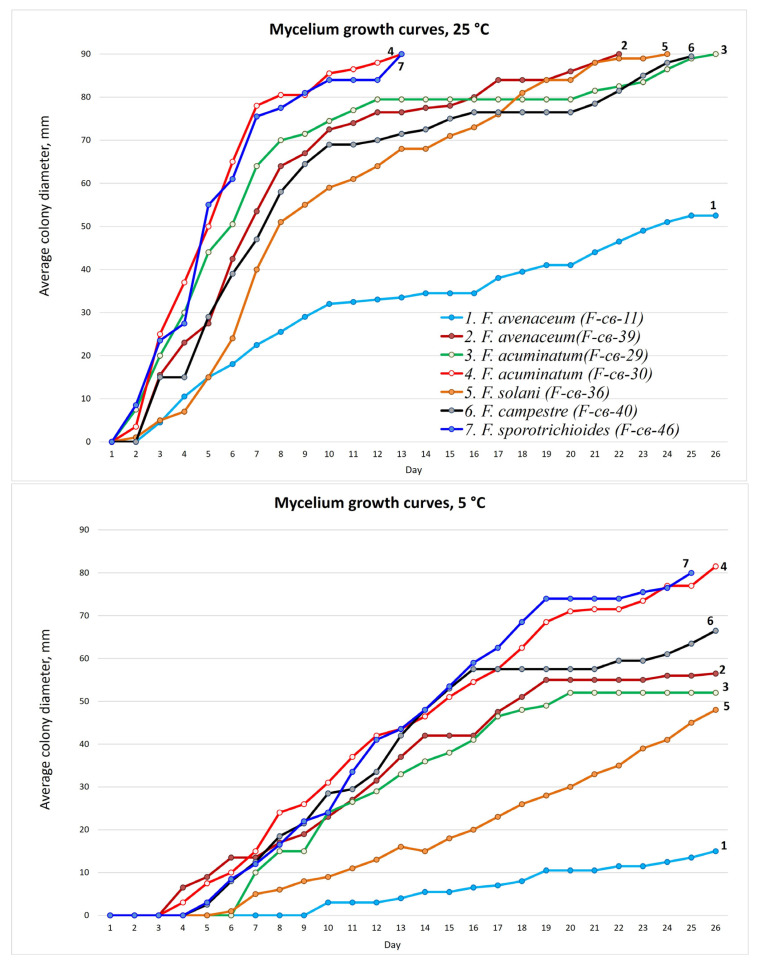
Mycelial growth of identified *Fusarium* species in the composition of the pathocomplex of storage rot of table beet roots at different temperatures (25 °C and 5 °C).

When grown on solid PDA medium at 25 °C, most defined strains of different *Fusarium* species showed a rapid onset of growth as early as day 1–2, followed by active exponential growth in the first 7–10 days and reaching a plateau of 75–90 mm by days 12–18 (Figure 8). The fastest-growing isolates were F-CB-30 of *F. acuminatum* and F-CB-46 of *F. sporotrichioides*, with an average colony growth rate of 0.7 cm/day and reaching a plate diameter of 90 mm by day 13. Isolate F-CB-11 of *F. avenaceum* was characterized by the slowest growth rate—0.2 cm/day; by day 26, the colony diameter was only 53 mm. The remaining isolates analyzed at 25 °C had an average colony growth rate of 0.34–0.41 cm/day and reached the plate diameter on days 22–26 of cultivation.

Considering that the second half of table beet storage occurs at 4–6 °C, we studied the growth of defined strains on PDA medium at 5 °C to potentially predict the dominant pathogenic *Fusarium* species in the storage rot pathocomplex. Results showed that at 5 °C, all analyzed species exhibited a distinct lag phase. Colony growth was virtually absent during the first 6–8 days. Afterward, very slow linear growth was observed, and by day 26, colony diameter was 1.5–2 times smaller than when cultured at 25 °C (Figure 8). The highest average growth rate at low temperatures was observed for isolates F-CB-30 of *F. acuminatum* and F-CB-46 of *F. sporotrichioides*—0.29 and 0.28 cm/day, with colony diameters on day 26 of 76 and 72 mm, respectively. Isolate F-CB-11 of *F. avenaceum* demonstrated the slowest growth rate—0.05 cm/day. The colony diameter of this isolate on day 26 was 13 mm.

**Figure 9:**
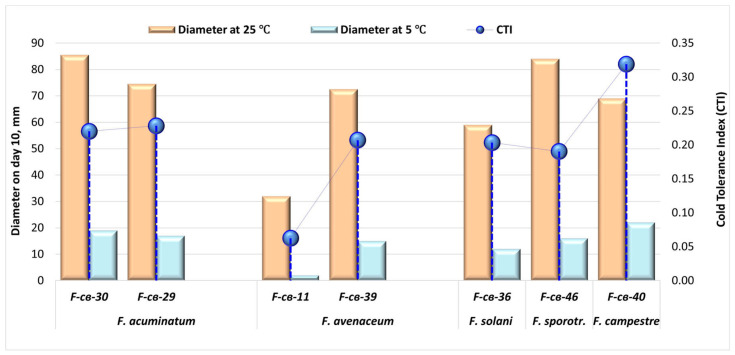
Colony diameter on PDA medium at different temperatures and the low temperature sensitivity index (CTI) of identified *Fusarium* species (day 10).

The sensitivity of isolates to low temperatures is most clearly shown in Figure 9, where significant differences are visible between isolates even within the same species, indicating intraspecific variability in low temperature tolerance. Based on the CTI value, isolate F-CB-40 of *F. campestre* was characterized by higher cold resistance (CTI 0.32), while isolate F-CB-11 of *F. avenaceum* was characterized by higher cold sensitivity (CTI 0.06). *F. acuminatum* isolates differed in growth rates at different temperatures, but all showed relative tolerance to low temperatures (CTI 0.22–0.23).

**Figure 10:**
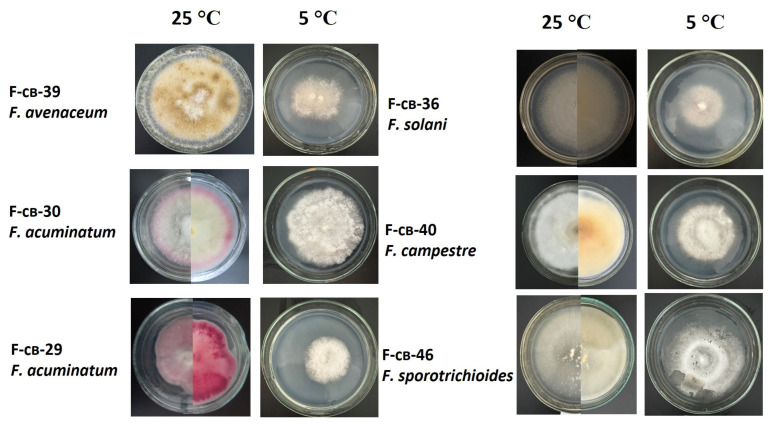
Appearance of colonies of representative strains of identified *Fusarium* species causing storage rot of table beet roots, at different cultivation temperatures (26 days).

On day 26 of cultivation, the analyzed isolates differed in mycelial structure and density, as well as pigment formation (Figure 10). At 25 °C, species-specific pigment formation was observed from days 7 (*F. solani*) to 19 (*F. acuminatum*). At 5 °C, colonies of all analyzed species did not produce pigments.

### 3.7. In Vitro Growth of Fusarium Species on a Natural Substrate (Beet Root Discs) at Different Temperatures

The most aggressive representative strains of the five identified species involved in the pathogenesis of storage rot were used to inoculate root disks from 13 table beet breeding lines. The inoculation procedure was performed with agar blocks at different temperatures: 5 °C (simulating root storage conditions) and 25 °C (simulating plant vegetation conditions). As a result, as in the experiment studying the pathogenic properties, it was shown that at 25 °C, *Fusarium* species differ in their aggressiveness against table beet roots depending on the host plant genotype. Under the conditions of this experiment, the species *F. avenaceum*, *F. campestre*, *F. sporotrichioides*, and *F. solani* were characterized by weak to moderate aggressiveness and did not differ statistically from each other (Table 6). The average volume of the affected area in case of inoculation with these species ranged from 5 to 21 mm^3^. *F. acuminatum* was the most aggressive species, with the average volume of the affected area of 663 mm^3^, ranging from 31 to 1760 mm^3^. In this respect, it was significantly different from other species.

As can be seen from Table 6, lines 208, 207, and 185 were characterized, on average, by a small affected area (15–22 mm^3^), including when infected with a highly aggressive *F. acuminatum* isolate, which allows us to consider them as potential sources of resistance to *Fusarium* root rot. Lines 184, 174, and 179, on the contrary, significantly exceeded the other analyzed hybrid combinations in terms of average volume of the affected area (282–363 mm^3^), which is determined by their susceptibility to *F. acuminatum*, while damage by other species was insignificant and corresponded to the average values of most lines. Line 209 stood out by the largest volume of the affected area when inoculated with *F. sporotrichioides* (50 mm^3^).

At 5 °C, the majority of lines demonstrated virtually no pathogen development when disks were infected with *F. avenaceum* and *F. sporotrichioides* isolates (lesion diameter 0–1 mm). When infected with *F. campestre*, *F. solani*, and *F. acuminatum*, a multiple reduction in volume of the affected area was also observed compared to 25 °C (average for all hybrid combinations 0.3–4.5 mm^3^) (Table 7).

**Figure 11:**
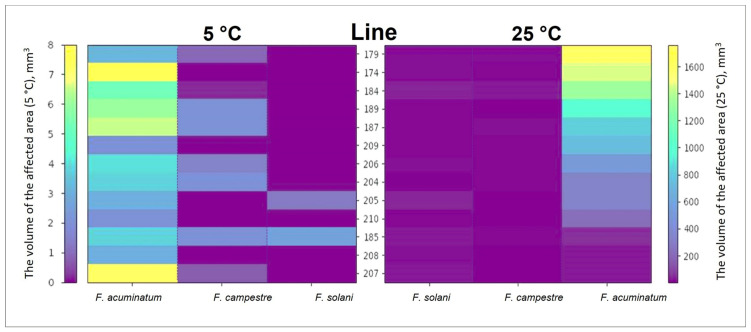
Heat map of the volume of the affected area of root cuttings (average of five replications) for hybrid combinations of table beet on the tenth day after artificial inoculation with isolates of *F. solani*, *F. campestre* and *F. acuminatum* (at 5 °C and 25 °C).

While *F. acuminatum* was poor growing in cold conditions, this species demonstrated the greatest aggressiveness. In the case of *F. acuminatum*, the volume of the affected area was significantly larger than that of other species (4.5 mm^3^ on average), and the analyzed lines also differed in the volume of the affected area, which varied in the range from 0.7 to 8.0 mm^3^. In this case, the average volume of the affected area of lines 174, 189, 185, 187, and 207 was significantly larger than that of the other lines (Table 7). The level of resistance of the lines was generally determined by their response to infection, depending on the temperature background (Figure 11).

### 3.8. The Effect of Pathogen Species, Temperature, Substrate Type, and Plant Genotype on the Development and Pathogenic Properties of Fusarium Fungi

**Figure 12:**
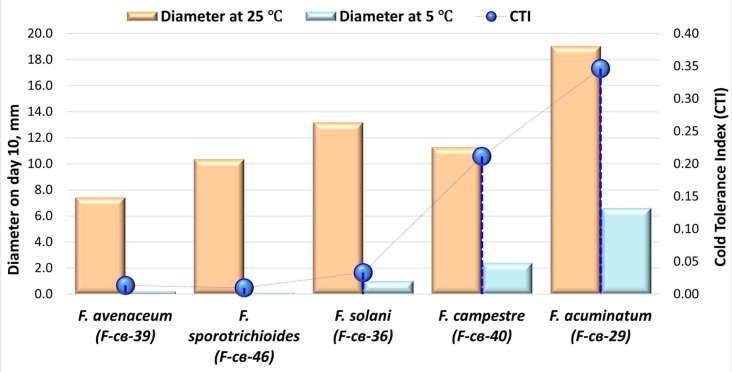
The diameter of the affected area of root disks at different temperatures and the cold tolerance index (CTI) of defined isolates of different *Fusarium* species (10th day).

The data obtained from inoculating root disks differ somewhat from the results of a study of the linear growth characteristics of colonies of identified isolates on an artificial nutrient medium (10 days). Thus, the decrease in *F. acuminatum* aggressiveness with decreasing temperature is less than the decrease in overall metabolism during growth on a nutrient medium, in contrast to *F. campestre*, the leader in cold resistance on an artificial medium. According to the cold tolerance index (CTI) values based on the average diameter of the affected areas of all lines (Figure 12), the studied *Fusarium* isolates were distributed in a different sequence—*F. acuminatum* (0.32) > *F. campestre* (0.21) > *F. solani* (0.03) > *F. avenaceum* and *F. sporotrichioides* (0.01), than when grown on a nutrient medium (Figure 10)—*F. campestre* (0.32) > *F. acuminatum* (0.22) > *F. avenaceum* (0.21) > *F. sporotrichioides* (0.19) > *F. solani* (0.20).

Temperature influences the interrelation between the linear growth rate of pathogens on a nutrient medium and a natural substrate. At the optimal temperature (25 °C), the correlation between these characteristics is very weak (r = 0.23). However, at a stressful low temperature (5 °C), the correlation is closer (r = 0.72), indicating common genetic or physiological mechanisms of cold adaptation for growth and infection processes. Moreover, the ranking of species by pathogenicity at 25 °C is generally maintained at 5 °C (r = 0.85).

In the studied pathosystem, the “pathogen species” factor is primary, determining up to 25–80% of the variation in damage severity. The dominant pathogen responsible for the main potential losses is *F. acuminatum*, whose aggressiveness on beet roots is orders of magnitude higher than that of the other studied species. The “temperature” factor is a key moderator: its contribution to the variation in linear colony growth on a nutrient medium reaches 80%. However, for pathogenic activity on root disks, its influence is mediated by the fungal species and a significant interaction between the “pathogen species × temperature” factors (10–12%).

Due to the complex nature of the resistance trait, the primary contribution of the host plant genotype is generally low (2–5%) and is revealed through interactions between factors, particularly in the triple relationship “plant genotype × pathogen species × temperature.” For example, plant genotype makes a significant contribution (up to 10%) in the case of the dominant pathogen *F. acuminatum*.

Correlation analysis did not reveal universal resistance to all *Fusarium* species. However, two groups of pathogens with cross-reactivity between genotypes were identified: *F. campestre*/*F. solani* (r = 0.75–0.85) and *F. avenaceum*/*F. sporotrichioides* (r = 0.62), indicating common resistance mechanisms. Susceptibility to the most aggressive species, *F. acuminatum*, is weakly correlated with response to other species.

## 4. Discussion

*Fusarium* species are known to affect beet plants worldwide at different stages of development, causing seed asphyxiation and seedling death [33,34], *Fusarium* wilt, root rot, and leaf and stem necrosis [35,36]. *Fusarium* root rot of beets, which develops during the post-harvest period during long-term storage, is of particular economic significance. This form of the disease is progressive and can lead to yield losses of up to 50% or more of the total weight of the stored product, making *Fusarium* root rot of beets a high phytopathological and economic risk [25,37,38]. Our long-term phytopathological monitoring of storage disease development in table beet roots confirmed this. *Fusarium* fungi have become a leading component of the pit rot pathocomplex in the last decade. In the Moscow region, root crop losses due to *Fusarium* rot can reach 58%, depending on the agroclimatic conditions of the year and the sample size [39]. The increase in Fusarium rot prevalence in individual years may be related to climatic factors, particularly an increase in average daily air temperature relative to long-term averages, agricultural practices, and an increase in the infection load in vegetable crop rotations.

Because research resources and investments are overwhelmingly concentrated on sugar beet, a strategic raw material for industrial processing, the most comprehensive information on the composition of the pathological complex of pathogens associated with *Fusarium* root rot is available specifically for this crop. Since the second half of the 20th century, about 20 species of *Fusarium* have been isolated and identified from affected parts of sugar beet plants [18,25,40,41,42].

However, currently, there is no up-to-date information on the species composition of the pathocomplex of *Fusarium* root rot during storage in table beet-growing regions of the Russian Federation, which undoubtedly complicates the development of a strategy for protection against this disease.

Due to the high intra- and interspecific variability of *Fusarium* morphology caused by the influence of genetic and environmental factors, phylogenetic analysis is an integral part of the correct identification of *Fusarium* species [43,44,45].

The presented study revealed significant changes not only in the species composition, but also in the succession of dominant *Fusarium* species that formed the currently existing pathocomplex of *Fusarium* root rot in table beets grown in the Moscow region of the Russian Federation. Molecular phylogeny using the TEF-1α and RPB2 genes, combined with morphological characterization, allowed us to establish the taxonomic affiliation and identify five main species that cause *Fusarium* root rot in table beet during storage: *F. acuminatum*, *F. avenaceum, F. campestre* (FTSC), *F. sporotrichioides* (FSAMSC) and *F. solani* (FSSC). Furthermore, *F. acuminatum*, *F. campestre*, and *F. sporotrichioides* were discovered for the first time on table beet roots in the Russian Federation. *F. acuminatum* was the dominant species in the analyzed pathocomplex (43% of the total isolates), while the percentages of other species were comparable to each other (within 14%). In Minnesota, USA, *F. acuminatum* was also one of the dominant species in the composition of *Fusarium* root rot of sugar beet. However, this study did not report its pathogenic potential on the host plant [42]. Our pathogenicity testing showed that this species exhibited the greatest aggressiveness in infecting beet roots. *F. solani* was the second most aggressive species, but this species exhibited the most pronounced cultivar-specific response. *F. sporotrichioides* and *F. campestre* isolates were characterized by moderate aggressiveness based on the average volume of the affected area, while *F. avenaceum* exhibited the weakest aggressiveness against the table beet roots of the analyzed varieties.

Another important aspect of our research was the study of the vital activity and pathogenic potential of identified *Fusarium* species under different temperature conditions, simulating the conditions of root formation during vegetation and storage. We were unable to find similar studies in the available scientific literature devoted to this issue in the context of *Fusarium* root rot. It is known that for the growth and development of most *Fusarium* species, the optimal temperature range is considered to be 25–30 °C [30], which is close to the temperature regime during the growing season of plants in the conditions of the Central and Southern regions of the Russian Federation. During root storage, a sharp increase in the spread of *Fusarium* rot is observed from January to March, when the storage temperature of table beet roots (in case of seed production) is increased from 2–3 °C to 4–6 °C. This leads to an accelerated spread of these facultative pathogens, which are known to most actively attack aging tissues [46], including those of table beet roots as a result of changes in their biochemical composition during storage.

To predict the harmfulness of species forming the *Fusarium* root rot pathocomplex, the linear colony growth of identified isolates of the detected species was studied in vitro on artificial nutrient medium (PDA) and a natural substrate (beet root discs) at contrasting temperatures of 5 °C and 25 °C. All species successfully attacked beet roots under optimal conditions (25 °C), with *F. acuminatum* occupying a leading position in both colony growth rate on the nutrient medium and diameter of the affected area of root discs. No such correlation was observed for other species, and it was also noted that immunological assessment of resistance to pathogens in this case may not always identify consistently resistant genotypes during cold storage, which is a stressful environment for pathogen development.

According to the cold tolerance index (CTI) value on PDA and root discs, the studied *Fusarium* isolates were also distributed differently: *F. campestre* (0.32) > *F. acuminatum* (0.22) > *F. avenaceum* (0.21) > *F. sporotrichioides* (0.19) > *F. solani* (0.20) and *F. acuminatum* (0.32) > *F. campestre* (0.21) > *F. solani* (0.03) > *F. avenaceum* > *F. sporotrichioides* (0.01), respectively. This confirms that in vitro studies of colony growth on artificial nutrient media cannot be directly extrapolated to assess the threat both during storage and during the growing season. Mandatory tests on a natural substrate (host plant) under different temperature backgrounds are necessary. The obtained results indicate that *F. acuminatum*, *F. campestre,* and *F. solani* are capable of infecting table beet roots across a wide temperature range. At the same time, *F. acuminatum* can be the dominant species in the complex of soil phytopathogens in areas of risky agriculture with a cool climate. *F. avenaceum* and *F. sporotrichioides* will cause most damage during the growing season at optimal temperatures. All identified *Fusarium* species were included in our breeding program to create resistant heterotic table beet hybrids.

In this case, it is of primary importance to expand knowledge about the resistance of varieties and hybrids to the most harmful species associated with *Fusarium* rot during the growing season and storage of roots, the introduction of which into production is the main component of the strategy for combating this disease [9].

In this regard, in parallel with studying the pathogenic properties, we assessed the resistance of the promising breeding material to five identified species involved in the pathogenesis of *Fusarium* root rot. It was found that the differentiation of genotypes into resistance groups to *Fusarium* root rot at a low temperature of 5 °C did not always coincide with that at 25 °C. This once again underscores the specificity of the interaction of factors in the complex “host genotype—pathogen species—environmental conditions” system.

Variation in the volume of the affected area is primarily explained by the pathogen species and temperature conditions, as well as their interactions. The influence of plant genotype manifests itself in a complex interaction with these two factors, especially under conditions optimal for pathogenicity. This confirms the need to consider abiotic environmental factors when screening and breeding for resistance. Low temperatures selectively and radically suppress some species (almost to zero) and only reduce the activity of others (*F. acuminatum*, *F. campestre*). The dependence of the degree of aggressiveness and harmfulness on external conditions indicates the importance of pathogen specialization.

The influence of the plant’s genotype manifests itself in a complex interaction with these two factors, especially under optimal conditions for pathogenesis. Immunological assessment of resistance under optimal pathogen conditions may not always identify valuable genotypes that exhibit consistent resistance under stressful conditions (particularly during storage).

When infected with the dominant, most aggressive species, *F. acuminatum*, the highest variation in the volume of the affected area was observed depending on the plant genotype at both temperature conditions. Therefore, this species can be considered the most objective differentiating factor when assessing the resistance of table beet roots to *Fusarium* root rot. When breeding table beet genotypes resistant to *Fusarium* rot under a wide range of conditions, it is necessary to conduct phenotypic evaluation at lower temperatures, focusing on the response specifically to *F. acuminatum*, supplemented by an assessment of resistance to a range of less aggressive, but regionally common, pathogenic *Fusarium* species.

It is important to note the alarming trend of widespread distribution of the identified species *F. acuminatum* in vegetable agrocenoses, which in our previous studies was also isolated from other vegetable crops in the Moscow region: from onion bulbs [47] and carrot roots [48] affected by rot during storage. Obtained isolates of this species were also characterized by high aggressiveness against their host plants. This may indicate that in four-field vegetable crop rotations (alternating crops: cabbage, carrots, onions, and beets) [49], which are most often used in vegetable farms, there is a real risk of this pathogen infecting various crops. In this regard, an important direction for further research is the study of the cross-pathogenicity of *F. acuminatum* and other *Fusarium* species isolated from table beet for other crops in order to find crops with a reduced risk of infection and to optimize vegetable crop rotations to reduce the infectious load in existing agrocenoses.

## 5. Conclusions

The study provided new insights into the biodiversity and pathogenicity of *Fusarium* fungi that cause Fusarium rot of table beet roots during storage in the conditions of the Moscow region in the Russian Federation. Molecular phylogeny using the *TEF-1α* and *RPB2* genes, combined with morphological characterization, allowed us to establish the taxonomic affiliation and identify five *Fusarium* species causing storage rot of table beet roots: *F. acuminatum*, *F. avenaceum*, *F. campestre* (FTSC); *F. sporotrichioides* (FSAMSC) and *F. solani* (FSSC). *F. acuminatum* was the dominant species in the analyzed pathocomplex (43% of the total number of isolates), while the percentage of other species was within 14%. The temperature sensitivity of the identified species at 5 °C and 25 °C was studied.

High pathogenicity of the *F. acuminatum* species, associated with this disease of table beet, for the host plant in the Russian Federation has been reported for the first time. It was established that the differentiating factor of genotypes for resistance to *Fusarium* rot under both temperature conditions is *F. acuminatum*, characterized by relative tolerance to low temperatures. In our study, breeding lines 208 and 210 demonstrated consistently low average damage values under both temperature conditions, which may indicate their genetically determined group resistance to the species *F. acuminatum*, *F. avenaceum*, *F. campestre*, *F. sporotrichioides* and *F. solani*, which are part of the *Fusarium* root rot pathocomplex.

The data obtained in the presented study are of great importance for the development of a strategy for the control of *Fusarium* fungi associated with storage rot of table beet roots, as well as other vegetable crops that are stored fresh for a long time, also in the case of seed production at low temperatures (vernalization).

## Figures and Tables

**Table 1:** Sequences of primers used for amplification of ITS, tef1 and rpb2 loci.

Primer	Sequence 5′-3′	Product Size (bp)	Ta (°C)
fRPB2-7cf RPB2-11ar	F: ATGGGYAARCAAGCYATGGG R: GCRTGGATCTTRTCRTCSACC	~900	52
ITS5 ITS4	F: GGAAGTAAAAGTCGTAACAAGG R: TCCTCCGCTTATTGATATGC	~550	56
EF-1 EF-2	F: ATGGGTAAGGARGACAAGAC R: GGARGTACCAGTSATCATG	~680	55

**Table 2:** Prevalence and average disease severity index of *Fusarium* rot in table beet samples during storage, distribution of affected roots by the type of localization of damage signs (Moscow region of the Russian Federation, 2018–2023).

Year	Prevalence in Sample, % (Min–Max)	Average Disease Severity Index, Points	Localization (% of Affected Roots)				
			Head	local Ulcers on the Surface	Inner Part	Tip	Entire Root
2018	3.0–77.0	2.2	50	16	6	3	25
2019	6.0–72.0	2.4	35	15	23	10	17
2020	5.0–34.0	1.8	20	60	10	5	5
2021	5.0–55.0	1.8	32	30	17	10	11
2022	2.0–48.0	2.2	30	37	11	5	17
2023	2.0–50.0	2.4	10	45	20	10	15
Average	-	2.1	30	33	15	7	15

**Table 3:** Collection of *Fusarium* isolates isolated from affected table beet roots in the Moscow region, which differ in their aggressiveness towards the host plant.

Isolate Code in 2024	A Year of Isolation	Localization		Aggressiveness Degree *	Average Volume of the Affected Area **, mm^3^
		Organ	Organ Part		
control					0 a
F-CB-52	2019	root	entire root	WA	23 ab
F-CB-39	2018	root	inner part		32 ab
F-CB-51	2019	root	tip		33 ab
F-CB-33	2020	root	tip		42 abc
F-CB-66	2023	root	inner part		45 abc
F-CB-64	2023	root	tip		52 abc
F-CB-22	2019	root	inner part		62 abc
F-CB-47	2019	root	inner part		65 abc
F-CB-40	2018	root	head	MA	79 abcd
F-CB-11	2018	root	inner part		79 abcd
F-CB-46	2019	root	entire root		82 abcd
F-CB-59	2020	root	inner part		89 abcd
F-CB-35	2018	root	tip		105 abcd
F-CB-49	2019	root	inner part		106 abcd
F-CB-30	2019	root	entire root		113 abcd
F-CB-62	2020	root	head		115 abcd
F-CB-32	2021	root	inner part		118 abcd
F-CB-44	2018	root	tip		118 abcd
F-CB-56	2019	root	head		119 abcd
F-CB-43	2018	root	inner part		121 abcd
F-CB-60	2020	root	inner part		121 abcd
F-CB-48	2019	root	head		133 abcd
F-CB-45	2018	root	head		144 abcd
F-CB-34	2018	root	tip	HA	166 bcd
F-CB-42	2019	root	entire root		186 cd
F-CB-41	2018	root	inner part		194 cd
F-CB-29	2021	root	head		224 d
F-CB-36	2018	root	head		348 e

Note: * WA—mildly aggressive; MA—moderately aggressive; HA—highly aggressive. ** The table shows the average values of the volume of the affected area for varieties of table beet Marusya, Krasny barkhat, when the cuttings of roots are infected with isolates; a–e: values with the same letter do not differ significantly with a probability of 95% according to the Duncan test.

**Table 4:** Micromorphological characteristics of identified *Fusarium* strains isolated from table beet roots during storage.

Characteristic	*F. campestre* (F-CB-40)	*F. sporotrichioides* (F-CB-46)	*F. avenaceum* (F-CB-39)	*F. solani* (F-CB-36)	*F. acuminatum* (F-CB-30)
Mycelium:					
growth pattern	radial with a dense center	radial with a sparse center	uniform or radial with rings	uniform creeping	radial with a sparse ring around a dense center
color	white	Gray–white	uneven gray-beige	light gray	uneven white with a pink edge
density	medium	low	high	low	medium
Stroma color	uneven beige-white	uneven light cream	uniform light brown	uniform light gray	slightly yellowish with a bright pink edge or intense dark pink
Microconidia:					
size, µm	11.8 ± 2.0 × 3.9 ± 0.2	9.1 ± 1.8 × 6.4 ± 0.9	13.6 ± 2.6 × 2.6 ± 0.4	9.1 ± 0.7 × 2.4 ± 0.1	4.1 ± 0.2 × 1.4 ± 0.1
septation	0–1	0–1	1–2	1	0–1
shape	oval	pyriform or oval	oval	oval, slightly curved	curved
Macroconidia:					
size, µm	28.2 ± 3.1 × 4.8 ± 0.4	19.2 ± 2.4 × 5.3 ± 0.8	47.1 ± 6.1 × 3.6 ± 0.9	14.5 ± 3.9 × 2.0 ± 0.5	9.8 ± 2.2 × 1.3 ± 0.2
septation	3	3	3–4	2–4	2–4
shape	fusiform or slightly curved	oval or slightly curved	straight or slightly curved	slightly curved	curved to crescent-shaped
Chlamydospores					
size, µm	absent	10.6 ± 1.2 × 10.4 ± 1.1	absent	3.3 ± 0.2 × 3.4 ± 0.4	absent
shape	absent	rounded	absent	rounded and round-oval	absent
abundance	absent	a lot, chains of 3–4 pieces	absent	a lot, one at a time, less often in pairs	absent

**Table 5:** Aggressiveness of *Fusarium* strains for edible roots of different varieties of table beet.

Variety	Volume of the Affected Area, mm^3^						
	Control (Agar Block)	*F. avenaceum* (F-CB-39)	*F. sporotrichioides* (F-CB-46)	*F. campestre* (F-CB-40)	*F. solani* (F-CB-36)	*F. acuminatum* (F-CB-29)	Average for the Variety
Krasny barkhat	0 Aa	19 Aa	49 Aa	64 Bab	248 Ac	164 Ac	109 a
Marusya	0 Aa	12 Aa	20 Aa	69 BCa	69 Aa	406 Ab	115 a
Dobrynya	0 Aa	49 Ba	180 Ba	78 Ca	817 Bb	303 Aa	285 ab
Lyubava	0 Aa	78 Ca	78 ABa	153 Da	78 Aa	870 Bb	251 ab
mean	0 Aa	39 A	81 AB	91 AB	303 BC	435 C	

Notes: A–D—values with the same capital letter in a column are not significantly different with a 95% probability according to Duncan’s test; a–c—values with the same capital letter in a row are not significantly different with a 95% probability according to Duncan’s test.

**Table 6:** The volume of the affected area of root cuttings (average of fifteen replications) of table beet hybrids on the tenth day after artificial inoculation with defined *Fusarium* strains (temperature 25 °C).

Genotype	Volume of the Affected Area, mm^3^						
	Control (Agar Block)	*F. avenaceum*	*F. campestre*	*F. sporotrichioides*	*F. solani*	*F. acuminatum*	Mean Value for Genotype
208	0 Aa	6 BCa	6 ABCa	13 Aa	18 Aa	33 Aa	15 a
207	0 Aa	7 Ca	7 ABCa	4 Aa	35 Aa	31 Aa	17 a
185	0 Aa	5 BCa	11 CDab	8 Aa	31 Ab	56 Ac	22 a
210	0 Aa	5 BCa	8 BCa	9 Aa	10 Aa	235 ABab	53 ab
204	0 Aa	2 ABa	9 BCa	3 Aa	8 Aa	335 ABb	71 ab
205	0 Aa	5 BCa	8 BCa	8 Aa	37 Aa	333 ABb	78 ab
206	0 Aa	2 ABa	11 CDa	7 Aa	19 Aa	522 ABb	112 ab
209	0 Aa	2 ABa	10 CDa	50 Ba	9 Aa	739 Bab	162 ab
187	0 Aa	4 ABCa	21 EFa	10 Aa	15 Aa	821 Bb	174 ab
189	0 Aa	4 ABCa	2 ABa	6 Aa	13 Aa	970 Bb	199 ab
184	0 Aa	7 Ca	24 Fa	8 Aa	41 Aa	1327 CDb	282 abc
174	0 Aa	3 ABa	9 BCDa	12 Aa	21 Aa	1452 CDb	299 abc
179	0 Aa	8 Ca	16 DEa	12 Aa	19 Aa	1760 Db	363 abc
Mean value for strain	0	5 A	11 A	12 A	21 A	663 B	

Notes: A–F—values with the same capital letter in a column are not significantly different with a probability of 95% according to Duncan’s test; a–c—values with the same capital letter in a row are not significantly different with a probability of 95% according to Duncan’s test.

**Table 7:** The volume of the affected area of root cuttings (average of fifteen replications) of table beet hybrids on the tenth day after artificial inoculation with defined *Fusarium* strains (temperature 5 °C).

Genotype.	Volume of the Affected Area, mm^3^				
	Control (Agar Block)	*F. solani*	*F. campestre*	*F. acuminatum*	Mean Value for Genotype
209	0 Aa	0 Aa	0 Aa	2.0 ABb	0.7 a
210	0 Aa	0 Aa	0 Aa	2.0 ABb	0.7 a
208	0 Aa	0 Aa	0 Aa	3.0 BCb	1.0 a
179	0 Aa	0 Aa	0.9 ABCa	3.2 BCb	1.4 a
205	0 Aa	1.3 ABab	0 Aa	3.0 BCb	1.4 a
184	0 Aa	0 Aa	1.2 BCa	5.2 CDEb	1.8 a
206	0 Aa	0 Aa	1.3 BCb	4.0 BCDc	1.8 a
204	0 Aa	0 Aa	2.0 Cb	3.8 BCDc	1.9 a
174	0 Aa	0 Aa	0 Aa	7.8 Fb	2.6 ab
189	0 Aa	0 Aa	2.0 Ca	6.0 DEFb	2.7 ab
185	0 Aa	2.6 Bab	2.0 Cab	3.8 BCDb	2.8 ab
187	0 Aa	0 Aa	2.0 Cb	6.5 EFc	2.8 ab
207	0 Aa	0 Aa	0.7 ABa	8.0 Fb	2.9 ab
Mean value for strain	0 A	0.3 A	0.9 A	4.5 B	

Notes: A–F—values with the same capital letter in a column are not significantly different with a 95% probability according to Duncan’s test; a–c—values with the same capital letter in a row are not significantly different with a 95% probability according to Duncan’s test.

## Data Availability

The original contributions presented in the study are included in the article; further inquiries can be directed to the corresponding author.
